# Parallel ICA identifies sub-components of resting state networks that covary with behavioral indices

**DOI:** 10.3389/fnhum.2012.00281

**Published:** 2012-10-11

**Authors:** Timothy B. Meier, Joseph C. Wildenberg, Jingyu Liu, Jiayu Chen, Vince D. Calhoun, Bharat B. Biswal, Mary E. Meyerand, Rasmus M. Birn, Vivek Prabhakaran

**Affiliations:** ^1^Neuroscience Training Program, University of WisconsinMadison, WI, USA; ^2^Medical Scientist Training Program, University of WisconsinMadison, WI, USA; ^3^The Mind Research NetworkAlbuquerque, NM, USA; ^4^Department of Electrical and Computer Engineering, University of New MexicoAlbuquerque, NM, USA; ^5^Department of Radiology, University of Medicine and Dentistry of New JerseyNewark, NJ, USA; ^6^Department of Medical Physics, University of WisconsinMadison, WI, USA; ^7^Department of Biomedical Engineering, University of WisconsinMadison, WI, USA; ^8^Department of Psychiatry, University of WisconsinMadison, WI, USA; ^9^Department of Radiology, University of WisconsinMadison, WI, USA

**Keywords:** resting state fMRI, parallel ICA, resting state networks, behavior

## Abstract

Parallel Independent Component Analysis (para-ICA) is a multivariate method that can identify complex relationships between different data modalities by simultaneously performing Independent Component Analysis on each data set while finding mutual information between the two data sets. We use para-ICA to test the hypothesis that spatial sub-components of common resting state networks (RSNs) covary with specific behavioral measures. Resting state scans and a battery of behavioral indices were collected from 24 younger adults. Group ICA was performed and common RSNs were identified by spatial correlation to publically available templates. Nine RSNs were identified and para-ICA was run on each network with a matrix of behavioral measures serving as the second data type. Five networks had spatial sub-components that significantly correlated with behavioral components. These included a sub-component of the temporo-parietal attention network that differentially covaried with different trial-types of a sustained attention task, sub-components of default mode networks that covaried with attention and working memory tasks, and a sub-component of the bilateral frontal network that split the left inferior frontal gyrus into three clusters according to its cytoarchitecture that differentially covaried with working memory performance. Additionally, we demonstrate the validity of para-ICA in cases with unbalanced dimensions using simulated data.

## Introduction

There has been a recent explosion of interest in the neuroscience community in identifying individual or group differences in intrinsic functional connectivity that correlate with specific behaviors or traits. To date, traditional analyses have been mostly univariate in nature. In traditional univariate fMRI analyses, individual voxels are assumed to be independent; that is, the variance of one voxel is not considered as an influence in the variation of any other voxel. Univariate methods of identifying relationships between different data modalities (i.e., data fusion) also share this limitation, as the relationship between modalities is considered independent at each voxel. More accurate, informative, and complex relationships between datasets can be identified by simultaneously considering all data in a multivariate and multimodal fashion (Calhoun et al., 2009). Such methods allow for variation of one variable, such as neuroimaging data, to inform variation of another, such as behavioral data. Furthermore, multivariate methods limit the number of statistical tests that are ultimately performed, as all relationships are tested together as opposed to being tested separately.

One such multivariate method of identifying resting state networks (RSNs) is Independent Component Analysis. ICA on resting state data can identify similar networks as other methods of analyzing resting state data (e.g., seed-based correlation). However, ICA does not require any *a priori* seed-region, and also considers both spatial and temporal information in indentifying RSNs (Calhoun et al., 2001; Beckmann et al., 2005). In addition to identifying components with unique temporal structures, the commonly used Group ICA Toolbox (GIFT) software also allows for back reconstruction of identified components into subject specific component maps. These maps can then used to compare the spatial and temporal characteristics of identified components between groups (Calhoun et al., 2001, 2009).

A relatively new method built upon the framework of ICA, called parallel independent component analysis (para-ICA), has been introduced as a multimodal data fusion tool that allows simultaneous multivariate analysis of two data types collected in the same subjects (Liu et al., 2008). One major benefit of para-ICA, along with the related method joint ICA, is that it is a completely data-driven second-level technique, requiring no *a priori* hypothesis regarding the relationship between modalities (Calhoun et al., 2009). The advantage of para-ICA over joint ICA is that para-ICA identifies the relationship between two fundamentally different types of data, whereas joint ICA is best suited for different data types that are assumed to modulate in the exact same manner (e.g., derived from the same spatial structure). Previously, para-ICA has been used to identify linear combinations of single nucleotide polymorphisms (SNPs) that covary with fMRI activations during an auditory oddball task or gray matter volume in schizophrenia patients (Liu et al., 2009; Jamadar et al., 2011), and spatial patterns of Amyloid-β that covary with rates of brain atrophy in MCI patients (Tosun et al., 2011).

In this study, we test the hypothesis that common RSNs can be spatially decomposed into sub-components that covary with specific behavioral profiles. It has been proposed that every functional network that is available during task performance is present during resting conditions as low frequency fluctuations in neural activity (Smith et al., 2009). Differences in functional connectivity in RSNs have been found to correlate with several different behavioral measures, including measures of intelligence (Song et al., 2008), reading competence (Koyama et al., 2011), risky behavior (Cox et al., 2010), working memory (Sala-Llonch et al., 2012), and spatial navigation (Wegman and Janzen, 2011). To our knowledge, no study has investigated whether sub-components of RSNs can be identified based on their covariance with different behavioral indices in a multivariate manner. Here, using simulated data we first demonstrate the ability of para-ICA to identify valid relationships between data modalities, even in cases in which the two data sets have unbalanced dimensionality. Finally, we use para-ICA to investigate the covariance between sub-components of several RSNs, including the attention, default, and frontal network, to linear combinations of a matrix of behavioral indices that require brain regions associated with these networks.

## Materials and methods

### Participants

Twenty-four healthy young adults (14 male; age 25 ± 0.67 years) provided informed consent to participate in three separate visits for this study. Twenty participants were right handed, one was left handed, and three were ambidextrous based on the Edinburgh Handedness Inventory. All aspects of this study were approved by the University of Wisconsin-Madison Health Sciences Institutional Review Board.

### Behavioral data acquisition

Participants completed a series of behavioral tasks and questionnaires outside of the scanner during a separate visit completed after the first scan visit, and inside the scanner during the second scan visit. Tasks performed outside the scanner included a computerized Stroop test (Stroop, 1935), a computerized Eriksen flanker task (Eriksen and Eriksen, 1974), digit and spatial forward and backward spans (Wechsler, 1997), Raven's Advanced Progressive Matrices (Raven et al., 1998), and a processing speed task (Wechsler, 2008). Tasks performed inside the scanner included a simple motor response task, a spatial working memory task, a verbal working memory task, and two combined spatial and verbal working memory tasks that our lab has previously used (Prabhakaran et al., 2000, 2011). Behavioral indices such as response time, accuracy, and rate were measured for each behavioral task as applicable (Table 1). Detailed explanations of each task used can be found below.

**Table 1:** **Displayed are the averages and SEMs across all subjects for the items included in the behavioral matrix for the para-ICA**.

**Task**	**Measure**	**Mean**	**SEM**
**Verbal WM**	RT	1045.13	50.77
	Accuracy	0.98	0.01
**Spatial WM**	RT	1124.37	43.8
	Accuracy	0.93	0.01
**Unbound WM**	RT	1298.54	47.55
	Accuracy	0.93	0.02
**Bound WM**	RT	1284	50.45
	Accuracy	0.96	0.01
Congruent	RT	1263.57	56.75
	Accuracy	0.95	0.01
Incongruent	RT	1406.05	57.48
	Accuracy	0.91	0.02
**Digit span**	Forward	11.5	0.5
	Backward	9.21	0.74
	Total	20.29	0.96
**Spatial span**	Forward	9.38	0.36
	Backward	9.42	0.36
	Total	18.79	0.52
**DSST**	Items/sec	0.74	0.03
**Finger tap**	RT	386.98	22.60
	Rate	2.86	0.21
**Flanker**	RT	502.07	9.82
	Accuracy	0.98	0.00
Congruent	RT	478.63	10.09
	Accuracy	0.99	0.00
Incongruent	RT	521.6	11.67
	Accuracy	0.97	0.01
Neutral	RT	499.43	11.53
	Accuracy	0.99	0.00
**Stroop**	RT	844.84	24.52
	Accuracy	0.97	0.01
Congruent	RT	826.45	23.45
	Accuracy	0.99	0.01
Incongruent	RT	863.92	26.75
	Accuracy	0.95	0.01
**Raven's APM**	Items	27.38	0.77

Handedness (20 R, 1 L, 3 Amb) scores from the Edinburgh Handedness Inventory, gender (14M/10F), and age (25 ± 0.67 years) for each subject were also included in the behavioral matrix for the para-ICA. RT, response time, measured in milliseconds. WM, working memory; DSST, digit symbol substitution task; APM, advanced progressive matrices.

#### Stroop task

All participants performed an in-house computerized Stroop test (Stroop, 1935) in which names of colors were presented on the screen for 1500 ms followed by 500 ms ITI. Participants were instructed to indicate the font color of the word by pressing the appropriate key marked with colored tape. For example, if the word “green” was presented in red font color, then the correct response would be to select the red taped key on the keyboard. This is an example of an incongruent trial. If the word “green” was presented in green font color then the correct response would be to select the green tapes key on the keyboard. This is an example of a congruent trial. Response time and accuracy were recorded for all trials, and the average response time and accuracy for all trials, congruent trials only, and incongruent trials only for each subject were entered into the behavioral matrix for the para-ICA.

#### Flanker task

For the computerized flanker task (Eriksen and Eriksen, 1974), an arrow and four distracters were presented on the screen for 1000 ms followed by 500 ms ITI. Subjects were instructed to focus on the arrow in the middle and press the arrow key pointing toward the same direction (left or right) on the keyboard. In congruent trials, all arrows pointed in the same direction (>>>>>). In incongruent trials, the distracters were arrows pointed in the opposite direction than the middle arrow (>><>>). In neutral trials, the middle arrow was flanked by four crosses (++>++). Average response time and accuracy for all trials, congruent trials only, incongruent trials only, and neutral trials only for each subject were each included in the behavioral matrix for the para-ICA.

#### Digit span

Forward, backward, and total digit span were recorded for each participant (Wechsler, 1997). In this task, increasingly longer strings of numbers were read aloud to participants. For the forward digit span, subjects were instructed to repeat the numbers in the same order as they were read to them. For the backwards digit span, subjects were instructed to repeat the numbers in reverse order that they were read. Tests were administered and scores calculated according to the WMS-III (Wechsler, 1997), with the total span measure equaling the sum of the scores on the forward and backward spans. Total, forward, and backward digit span for each subject were included in the behavioral matrix for the para-ICA.

#### Spatial span

Forward, backward, and total spatial span were recorded for each participant (Wechsler, 1997). In this task, increasingly longer sequences of blocks on a standard board were touched by the experimenter. For the forward spatial span, subjects were instructed to repeat the sequence in the same order as they were touched by the experimenter. For the backward spatial span, subjects were instructed to repeat the sequence of touched blocks in reverse order than they were pressed by the experimenter. Tests were administered and scores calculated according to the WMS-III (Wechsler, 1997), with the total span measure equaling the sum of the scores on the forward and backward spans. Total, forward, and backward spatial span for each subject were included in the behavioral matrix for the para-ICA.

#### Verbal working memory

Participants completed a verbal working memory task inside the scanner. For each trial, three upper case letters (excluding vowels and “L”) were presented in the center of a back projected screen for 2 s. Following a delay period of 6 s a single lower case letter was presented in the center of the screen for 2 s. Trials were separated by varying ITI. Changing the case of the letter ensured subjects were encoding the information verbally. Subjects responded using a MR-safe button pad whether or not the lower case letter was one of the three letters they had previously seen. Average response time and accuracy for each subject were included in the behavioral matrix for the para-ICA.

#### Spatial working memory

In the spatial working tasks, three locations indicated by parentheses presented around an imaginary clock face case letters were presented in the center of a back projected screen for 2 s. Following a delay period of 6 s a single location was presented on the screen for 2 s. Trials were separated by varying ITI. Subjects responded using a MR-safe button pad whether or not that location was one of the three locations they had previously seen. Average response time and accuracy for each subject were included in the behavioral matrix for the para-ICA.

#### Bound spatial and verbal working memory

Subject also performed two combined verbal and spatial working memory tasks designed to identify the neural substrates of working memory feature binding (Prabhakaran et al., 2000, 2011). In the bound task three upper case letters were presented in three different locations around an imaginary clock face on a back projected screen for 2 s. Following a delay period of 6 s a single lower case letter at a single location around the clock face was presented on the screen for 2 s. Trials were separated by varying ITI. Subjects responded using a MR-safe button pad whether or not *both* the letter and location were included in the previous screen. However, subjects were informed that the letter and location did not necessarily had to have been paired together initially in order for the trial to be a “match” trial. These are examples of incongruent match trials. In congruent match trials the letter and location presented in the retrieval phase were also paired together in the encoding phase. Average response time and accuracy for congruent trials, incongruent trials, and all trials from each subject were used as input to the behavioral matrix for the para-ICA.

#### Separate spatial and verbal working memory

In the separate, or unbound, working memory task, three upper case letters were presented in the center of the screen and three different locations indicated by parentheses were presented on an imaginary clock face around the center of the back projected screen for 2 s. Following a delay period of 6 s a single lower case letter at a single location around the clock face was presented on the screen for 2 s. Trials were separated by varying ITI. Subjects responded using a MR-safe button pad whether or not *both* the letter and location were included in the previous screen. Average response time and accuracy on all trials for each subject were used as input to the behavioral matrix for the para-ICA.

#### Digit symbol substitution task

Participants also completed the digit symbol substitution task (Wechsler, 2008). In this task, subjects were given 120 s to write the appropriate symbol associated with each specific number based on a key presented at the top of the page. The number of symbols corrected drawn per second for each subject was included in the behavioral matrix for the para-ICA.

#### Motor task

During the scanning session participants performed a simple block design motor task in which subjects were instructed to sequentially and repeatedly press four buttons on a MR-safe button pad with their four fingers (digits 2–5) when the word “Tap” appeared on the screen, starting with their index finger. Subjects were to stop and rest when the word “Rest” appeared on the screen. Twenty second long “Rest” and “Tap” blocks were alternated for 3 min (5 “Rest” blocks, 4 “Tap” blocks). The average response time for each button press and the number of total presses per second from each subject were included as variables in the behavioral matrix for the para-ICA.

#### Raven's advanced progressive matrices

Sets 1 and 2 of Raven's advanced progressive matrices, a canonical reasoning task, were completed by each subject (Raven et al., 1998). The 12 questions of set 1 were given as an untimed practice session, and each question answered incorrectly was reviewed and explained by the experimenter. Following this, subjects were given 40 min to complete the 36 questions in set 2. The total number of correctly answered questions from set 2 for each subject was entered into the behavioral matrix for the para-ICA.

### Resting state fMRI acquisition

Each participant completed nine, 10-min resting state scans, six of which were collected in the first visit and three were collected during a visit at least two months after the first visit. Of these nine scans, three were collected with the participants instructed to have their eyes closed, three were collected with the participants instructed to have their eyes open, and three were collected with the participants instructed to fixate their gaze on a fixation cross projected to the center of a MR-safe screen. For all resting scans, subjects were instructed to remain calm, still, and awake. Resting state scans were collected on a 3T MRI scanner (GE Healthcare, Waukesha, WI) using gradient-echo echo-planar imaging with the following parameters: TR = 2.6 s, TE = 22 ms, field of view = 22.4 cm, flip angle = 60°, 40 sagittal slices, acquisition matrix = 64 × 64, 3.5 mm isotropic voxel size, 231 time-points. T1-weighted anatomical images were collected at each scan visit using a FSPGR BRAVO (TR = 8.132 ms, TE = 3.18 ms) over a 256 × 256 matrix and 156 slices (flip angle = 12°, FOV = 25.6 cm, slice thickness = 1 mm).

### Resting state fMRI preprocessing

The first three time points of each scan were removed, and images were slice time corrected and motion corrected using AFNI (Cox, 1996). Participants' anatomical scans were registered to each functional scan and then normalized to standard MNI space in SPM8. This transformation was then used to map the functional scans to MNI space with a resampling to 3 × 3 × 3 mm^3^. Functional scans were then spatially smoothed with an 8 mm^3^ full width half maximum isotropic Gaussian kernel in AFNI.

Head motion has been shown to significantly affect resting state connectivity (Power et al., 2012; Satterthwaite et al., 2012; Van Dijk et al., 2012). Therefore, a two-way repeated measures analysis of variance, with factors of eye condition (close, fixate, open) and scan time (1, 2, and 3), was performed. The dependent variable of head motion was calculated as the square root of the sum of squares of the derivatives of the six motion parameters that were output during volume registration (Jones et al., 2010). There was no effect of eyes [*f*_(2, 46)_ = 0.01, *p* = 0.99] or scan time [*f*_(2, 46)_ = 0.88, *p* = 0.42], and the interaction of eyes and scan time was also not significant [*f*_(4, 92)_ = 0.66, *p* = 0.62].

### First-level analysis: group independent component analysis

**Figure 1:**
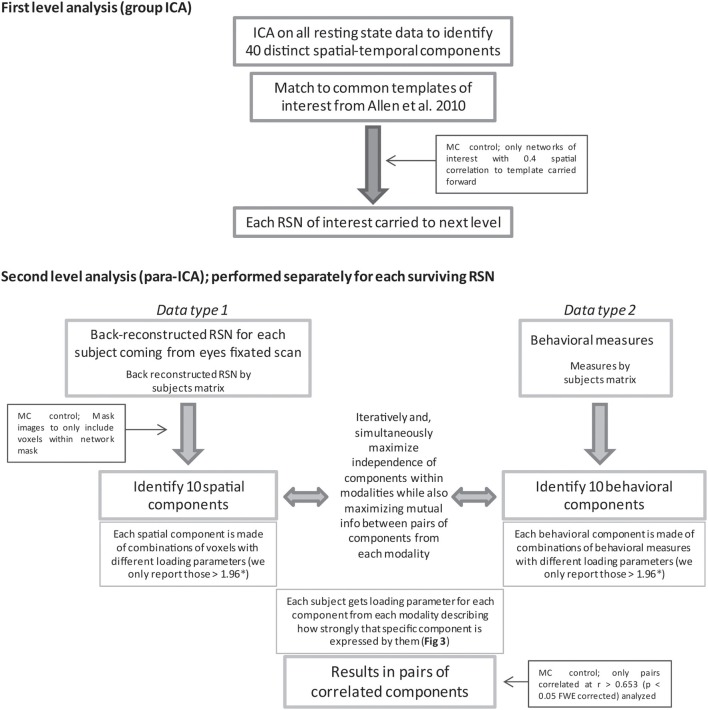
**Displayed is a flow chart of the methods used for this study.** MC and ^*^ indicate where steps were taken to limit multiple comparisons (MC). For in depth description of para-ICA analysis see the following papers (Calhoun et al., 2009; Liu et al., 2009).

An overview of the different analysis steps performed for this study can be seen in Figure 1. Before para-ICA can be run, a first level analysis identifying RSNs is necessary. In order to do so, group ICA was performed using temporal concatenation as implemented in the GIFT 1.3i program (Calhoun et al., 2009). Group ICA, as implemented in the GIFT program, is a completely data-driven method that can be used to identify spatially distinct, temporally coherent components from resting state data. One attractive feature of this program is that it provides back-reconstruction steps that produce subject, or session, specific spatial maps for each independent component extracted at the group level. This allows the spatial extent of each network to vary across subjects or sessions making post-analysis comparisons of network extent possible.

For the first level analysis of the resting state data, all 216 scans encompassing each resting state eye condition (open, closed, fixate) were entered into the group ICA. Although there may be subtle differences in some networks due to eye condition, all scans were included in order to obtain more robust ICA results. Any subtle differences that might occur due to resting scan instructions should be accounted for in the back reconstructed components. The entire dataset of 216 resting state runs was masked to include only brain structures, temporally concatenated, and two rounds of Principal Component Analyses (PCA) were performed prior to ICA to reduce the dimensionality of the data. Forty independent components were estimated using the Infomax algorithm to maximize the spatial independence of the components (Obradovic and Deco, 1998). The number of components to be estimated was ultimately selected based on the correspondence of the resulting components to known templates of RSNs. Five iterations of the ICA were performed via the ICASSO function to verify consistency of the components The ICASSO results indicated very high stability of the estimated components (average Iq = 0.97 ± 0.007 SD). Back-reconstructed spatial maps representing each component coming from each of the nine resting state scans for every subject were created using the GICA3 back-reconstruction algorithm in the GIFT software.

**Figure 2:**
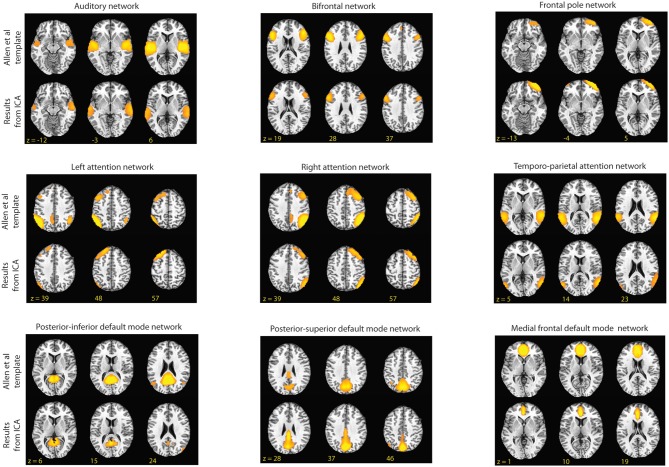
**Shown above are the RSNs identified by Allen et al. and the component from our group ICA that had the highest spatial correlation to the Allen et al. templates (2011).** In this figure, for the templates the threshold is set at *t* = 45 and for the matching components from our data the threshold is set at *z* = 3.0.

The resulting group components were then spatially correlated at a *z*-score threshold of 3.0 (*p* < 0.005) with templates of established RSNs provided online by the developers of the GIFT program at a threshold of *t* = 35.5 (*t*-value based on suggestion of template providers; Allen et al., 2011). In their original study, Allen et al performed group ICA on over 600 subjects and identified 28 components as being RSNs. These templates were used to confirm that the networks carried over for our para-ICA in the second level analyses are consistent with established common RSNs. In addition, the identification of common networks in our data provides verification that the number of independent components we estimated for the group ICA was appropriate. We limited our investigation to *a priori* selected networks in order to limit the total number of comparisons performed. For this study, we were interested in the attention networks, the default networks, and the frontal networks (Figure 2) as these networks involve brain regions thought to be responsible for many of the behavioral tasks performed by our subjects (Corbetta and Shulman, 2002; Koechlin and Summerfield, 2007; Buckner et al., 2008). In addition, we identified the auditory network as a negative control, with the hypothesis that variations in this network at rest should not correlate with our behavioral tasks.

Only components that spatially correlated with the provided templates at a correlation coefficient of 0.4 (*p* < 0.001) or greater were considered in order to limit our analyses to statistically significant correlations and moderate-to-strong correlations. This identified nine RSN's: a left frontal-parietal attention network, a right frontal-parietal attention, a bilateral temporo-parietal attention network, an auditory network, a posterior–superior component of the default mode network consisting of the precuneus, a posterior–inferior component of the default mode network consisting of the posterior cingulate, a medial frontal default mode network component, a bilateral frontal network, and a frontal pole network (Figure 2). The average dynamic range of these networks was 0.041 Hz and the average power ratio was 13.54. These networks were carried out for second-level para-ICA analyses.

### Second-level analysis: parallel independent component analysis

**Figure 3:**
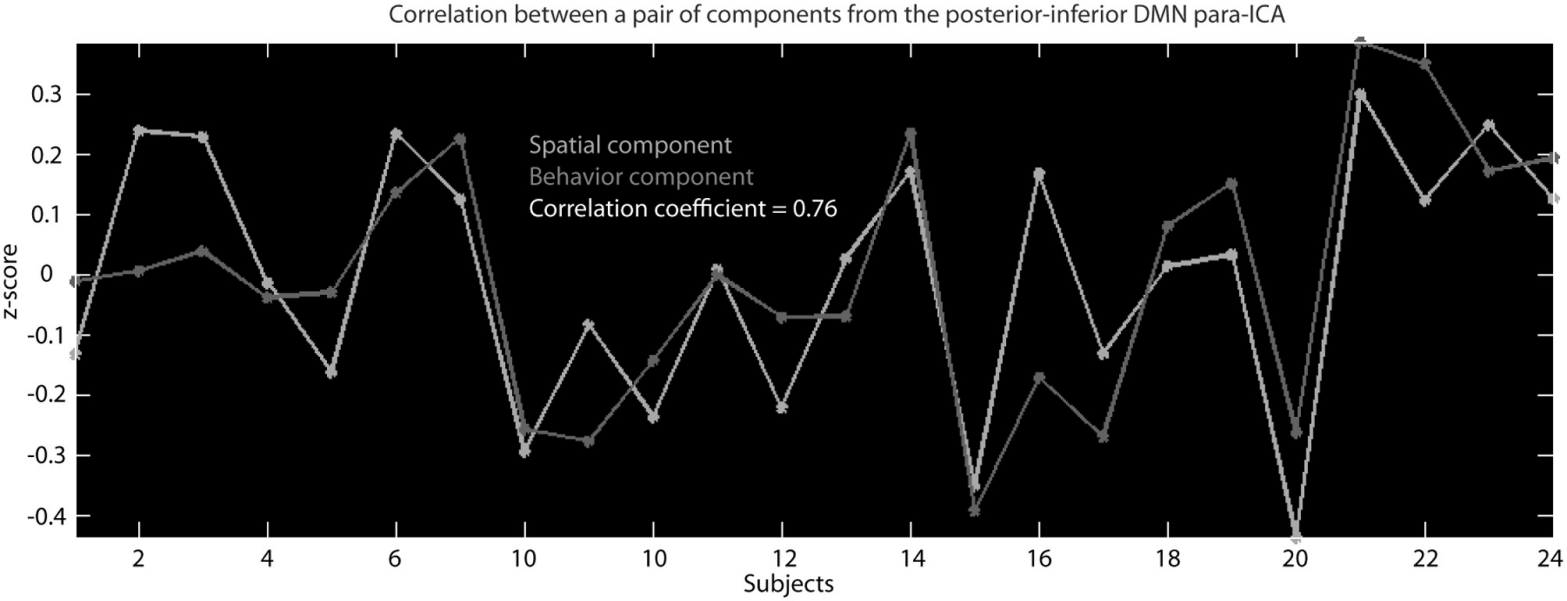
**This is an example of the correlation across subjects between the *z*-scaled loading parameters for a behavioral component and the *z*-scaled loading parameters of a spatial component that resulted from the para-ICA run for the posterior–inferior default mode network**.

Separate parallel ICA (para-ICA) analyses were carried out for each of the nine RSNs identified in the group ICA using the Fusion ICA Toolbox (http://icatb.sourceforge.net). Para-ICA is a second-level analysis that allows investigation of cross information between two different data types (Liu et al., 2009). Essentially, two ICAs are run simultaneously, one on the subject specific back-reconstructed RSN and one on the matrix of behavioral measures, with a term in each mixing matrix that describes the relationship between the two ICAs (Figure 1). The two un-mixing matrices are iteratively updated while the components from each modality with the highest correlation are selected and used to modify the de-mixing matrix until a stopping criteria is reached. This process results in a number of components for each data type that are differentially expressed in each subject which is quantified by a loading parameter per component for each subject. The variation of the expression (loading parameter) of a single component for one data type is correlated across subject with the expression of a single component from the second data type, resulting in pairs of correlated components (Figure 3) from each data modality (Liu et al., 2008; Calhoun et al., 2009).

There are three parameter settings within the para-ICA software that influence how the correlations between the two modalities are derived. The *constrained connection* parameter is the level of correlation between modalities at which the para-ICA selectively updates the de-mixing matrix. The *constrained components* parameter is the maximum number of paired components that can be updated based on the constrained connections threshold. Finally, the *endurance* parameter is the maximally allowed descending trend of entropy. More details regarding these parameters can be found elsewhere (Liu et al., 2008). Here, we use the default values of 0.3, 3, and −1e-3, respectively, for these three parameters.

Para-ICA allows for one data point entry for each modality. Here, the RSN data entered into each para-ICA was derived from the first functional scan during the visit where subjects were instructed to fixate their gaze on a fixation cross as this method is the most consistent version of the task, preventing subjects from random visual stimuli (compared to eyes open) and also has the lowest likelihood of subjects falling asleep (compared to eyes closed). For the other data type, a total of 39 behavioral and demographic measures, such as response time, accuracy, gender, and handedness were provided as input. Both the back-reconstructed component maps and the behavioral indices were converted to *z*-scores prior to para-ICA to ensure that the scale of each was comparable.

PCA was performed on each data set separately to approximate the number of components to estimate for each data type. Approximately 90% of the variance was explained by retaining 10 components for both the behavioral data and each resting state component. Therefore, 10 components were extracted for each data type. Each para-ICA was repeated 20 times to ensure consistency of the components. For each para-ICA, analysis of each RSN was limited to areas within a mask of that particular RSN at a threshold of *z* > 1.96 (*p* < 0.05) to limit our search to areas that contributed strongly to the RSN.

### Simulations

The primary goal of this research was to identify sub-components of common RSNs (high dimension data) that specifically covary with sub-components of behavioral indices (low dimension data). To alleviate potential concerns about the performance of para-ICA in the null case in which no relationship exist between data modalities, and to assess the robustness of para-ICA in scenarios with unbalanced data dimensions, simulations were performed. For the simulations a 100 sample dataset was created. To reflect the unbalanced dimensions the fMRI data were simulated to span 10,000 voxels and involve 8 independent components, while the behavioral data were simulated to consist of 5 variables and involve 4 independent components. Thus, the resulting dimension was 100-by-10,000 for the simulated fMRI data and 100-by-5 for the simulated behavioral data. The two modalities were connected with different levels of randomly ranging correlations that served as the ground truth. Para-ICA was then applied to the simulated dataset and the results compared with the ground truth to investigate the use of para-ICA in the null scenario of no relationship between data modalities. Each simulation was run 100 times, and default para-ICA parameters were used.

## Results

### Simulations

**Figure 4:**
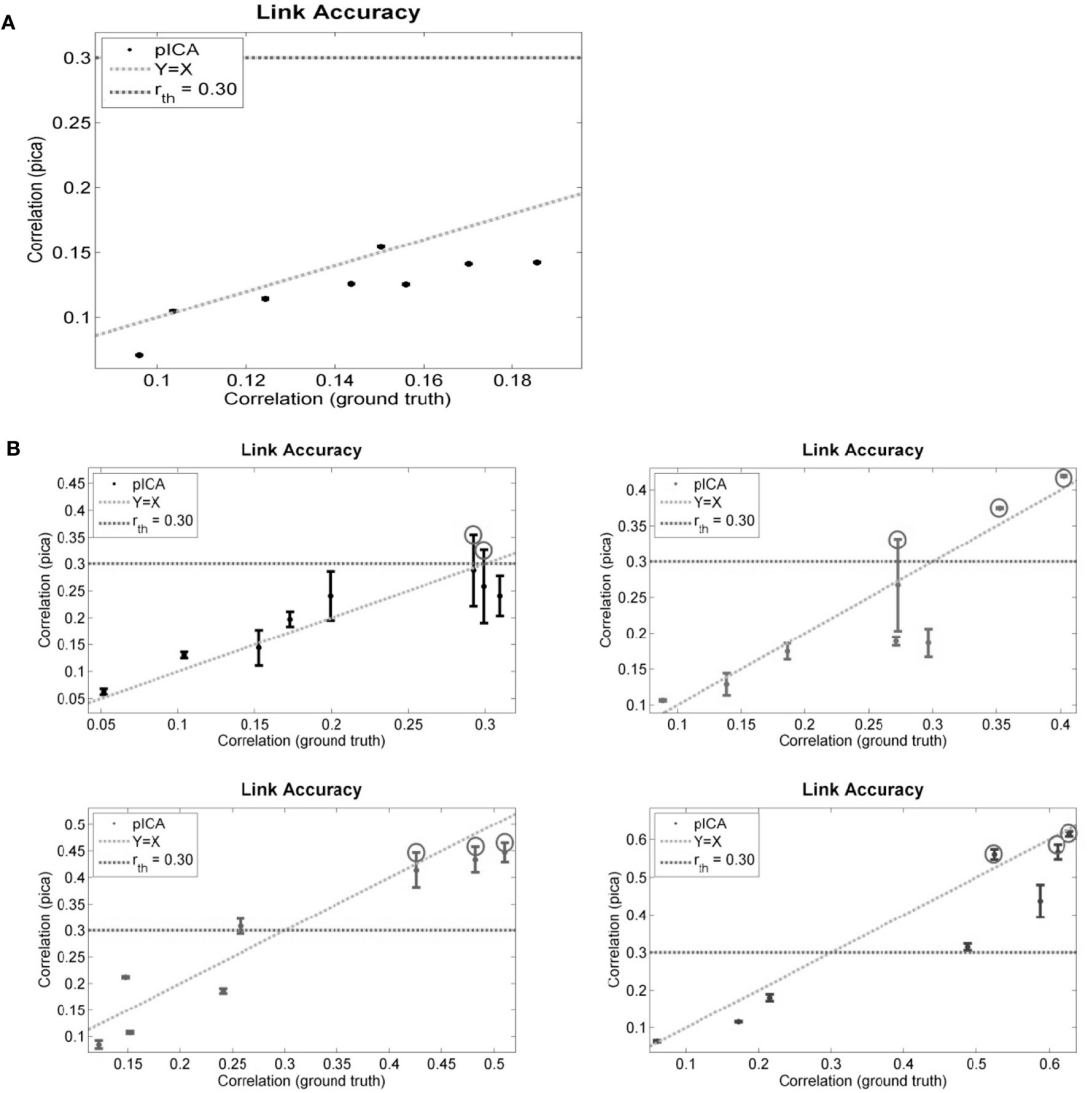
**Displayed are the simulated data results comparing the correlations derived from para-ICA to the ground truth correlations in data with highly unbalanced data dimensions in cases of sub-threshold correlations between data modalities (A) and in cases with varying supra-threshold correlations between modalities (B).** The dotted horizontal line reflects the constrained connections parameter (r_th) and the dotted diagonal line reflects a perfect match between the ground truth and the para-ICA derived correlations. Para-ICA treats data-modalities with only sub-threshold correlations as having no inter-modal relationship (as seen in **A**). When the correlation between pairs of components exceeds r_th, these components are updated in the para-ICA algorithm (as circled in **B**). A maximum of three sub-threshold pairs can be updated in the para-ICA framework. Error bars represent standard deviation based on 100 iterations. Note that error bars of **(A)** overlap with the data points and are hard to see due to low standard deviations.

The performance of para-ICA on simulated data in several scenarios of ground truth correlation between components is displayed in Figure 4. For each graph, the highest correlation between each of the eight simulated fMRI components and any one of the four simulated behavioral components is plotted against the ground truth correlation between that pair of components. The dotted horizontal line reflects the constrained connections parameter (r_th) used, and any component pair with correlation above r_th is circled, indicating that this pair (maximum three pairs) was updated based on the correlation constraint (see “Materials and Methods”). Figure 4A illustrates the performance of para-ICA in cases in which there are no inter-modal correlations that exceed r_th, resulting in the acceptance of the null hypothesis that the data modalities are not connected. Figure 4B summarizes the performance of para-ICA in cases of varying supra-threshold (r_th) correlations between modalities.

At the default parameter settings para-ICA only enhances the correlations of the top three pairs of components whose natural correlations are higher than 0.3. In null scenarios where no correlations exceed this threshold para-ICA essentially works as two separate ICAs. When the true correlation is near the 0.3 threshold, a larger deviation of derived correlations is seen due to the para-ICA correlation-based updating of the de-mixing matrix on pairs just above the threshold, but not on those just below the threshold. At higher true correlations, the para-ICA performs relatively precisely. These simulations demonstrate the validity of para-ICA in identifying correlations between components from two data modalities in cases of unbalanced data dimensionality.

### Resting state and behavioral data

**Figure 5:**
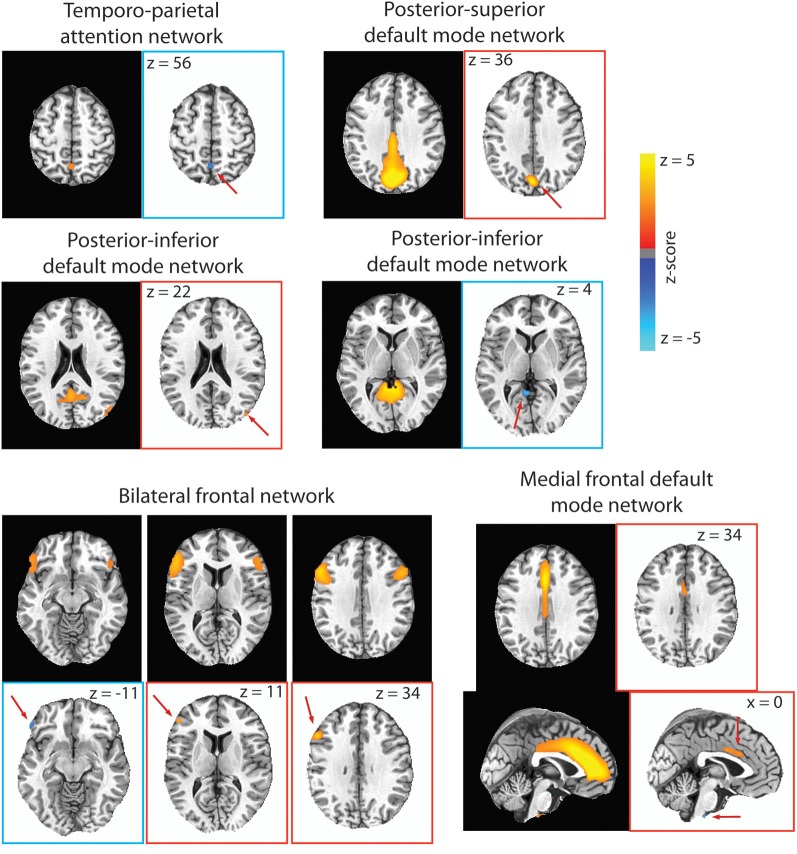
**For each RSN that had significant para-ICA correlations, the RSN from our group ICA is displayed on black background, while the spatial component resulting from the para-ICA is displayed on white background.** Spatial components with blue borders represent components with negative loadings while those with red borders represent components with positive loadings. Red arrows are included to direct the reader to the spatial components. Only voxels with loading parameter *z* > 1.96 (*p* < 0.05) are shown.

To control for multiple comparison, only components with an absolute correlation coefficient greater than 0.653 are reported (*p* < 0.05, Bonferonni FWE correction). For the spatial components, only clusters greater than 100 mm^3^ in volume (at least four contiguous voxels) are reported (Figure 5). Four of the nine para-ICA analyses did not have any IC pairs meet this criterion. These include the auditory network, the frontal pole network, and the two lateralized attention networks.

The para-ICA procedure produces pairs of components from each data type (Table 2). The components from the RSN are spatial maps of *z*-scaled loading parameters at each voxel. The components coming from the behavioral matrix consists of *z*-scaled loading parameters for each behavioral measure. For visualization, only voxels with a *z*-scaled loading parameter *z* > 1.96 (*p* < 0.05) are displayed. Likewise, only behavioral indices with a *z*-scaled loading parameter *z* > 1.96 (*p* < 0.05) are presented and discussed.

**Table 2:** **Displayed above are the RSNs that had pairs of components from the para-ICA that were significantly correlated (*r* > 0.653)**.

**RSN**	**“+” sub-component areas**			**“−” sub-component areas**			**Behavior**	**Behavior *z*-score**	**CC**
	**Area**	**MNI coordinates**	**Volume mm**^3^	**Area**	**MNI coordinates**	**Volume mm**^3^			
Temporo-parietal attention	–	–	–	R precuneus	[3, −55, 58]	351	Stroop Con. RT	2.55	−0.67
							Stroop Incon. RT	−2.13	
Posterior–superior default mode	Cuneus	[0, −77, 38]	2133	–	–	–	Spatial RT	−3.75	−0.67
							Bound Incon. RT	−2.02	
Posterior–inferior default mode	R mid. occ. gyr. BA 19	[48, −82, 22]	108	–	–	–	Unbound RT	−2.9	0.759
							Stroop Con. RT	2.19	
Posterior–inferior default mode	–	–	–	L vermis	[−3, −49, 4]	513	Stroop Incon. RT	4.13	−0.697
							Stroop RT	2.5	
Medial frontal default mode	M cing. BA 24	[0, 2, 34]	783	R brainstem	[3, −28, −53]	243	Verbal RT	−4.02	−0.723
							Unbound RT	2.23	
Bilateral frontal	L inf. front. oper. BA 44	[−51, 23, 34]	999	L inf. front. orb. BA 47	[−51, 38, −11]	135	Bound RT	−3.9	−0.698
	L inf. front. tri. BA 45	[−48, 41, 13]	324						

Only behaviors and spatial clusters with loading parameter z > 1.96 (p < 0.05) are displayed. In addition, only spatial clusters with a minimum 100 mm^3^ volume were included for this analysis in order to limit analysis of extremely small clusters (must have at least four contiguous voxels).

### Temporo-parietal attention network

A significant negative correlation (CC = −0.67; Table 2) was found between a sub-component of the temporo-parietal attention (Figure 5) network consisting of a 351 mm^3^ cluster located in the precuneus with a negative loading parameter and a behavioral component consisting of response time in congruent (*z* = 2.55) and incongruent (*z* = −2.13) trials of the Stroop test. Less connectivity of this area of the precuneus to the temporo-parietal attention network negatively covaried with response time in congruent Stroop trials, but positively covaried with greater response time in incongruent Stroop trials.

### Posterior–superior default mode network

For the posterior–superior default mode network, a significant negative correlation (CC = −0.67; Table 2) was found between a positively weighted 2133 mm^3^ cluster (Figure 5) located in the cuneus and a behavioral component consisting of response time in the spatial working memory task (*z* = −3.75) and response time in incongruent trials of the bound working memory task (*z* = −2.02), which requires simultaneous retention of both spatial and verbal information. This indicates that greater connectivity of this region of the cuneus to the posterior–superior default mode network negatively covaries with faster response times in two separate tasks involving spatial working memory.

### Posterior–inferior default mode network

A significant positive correlation (CC = 0.759; Table 2) was found between a behavioral component that includes response time in the unbound working memory task of both spatial and verbal information (*z* = −2.90) and response time in congruent trials of the Stroop task (*z* = 2.19) to a 108 mm^3^ cluster located in the right middle occipital gyrus with positive loading (Figure 5). Thus, greater connectivity of the right middle occipital gyrus to the posterior–inferior default mode network positively covaries with slower response time in congruent trials of the Stroop task and faster response time in the unbound spatial and verbal working memory task.

A significant negative correlation (CC = –0.697) was found between a 513 mm^3^ cluster in the cerebellum (Figure 5) and a behavioral component consisting of response time in both incongruent Stroop trials (*z* = 4.13) and all to the average response time to all Stroop trial types (*z* = 2.50). This can be interpreted as less connectivity of this area in the cerebellum to the posterior–inferior default mode network negatively covaried with slower response time in incongruent trials of the Stroop task and in all trials in the Stroop task.

### Medial frontal default mode network

A significant negative correlation (CC = –0.73; Table 2) was found between a behavioral component including response time in the verbal working memory task (*z* = −4.02) and response time in the unbound working memory task involving both spatial and verbal information (*z* = 2.23) to a 783 mm^3^ cluster in the mid-cingulate cortex and a 243 mm^3^ cluster in the brainstem (Figure 5). Greater connectivity of mid-cingulate cortex and less connectivity of the brainstem to the medial frontal default mode network is negatively covaried to faster response time in the verbal working memory task and slower response time in the unbound working memory task, which requires the simultaneous retention of separate spatial and verbal information.

### Bilateral frontal network

A significant negative correlation (CC = −0.70) was found between a behavioral component including response time in the bound working memory task (*z* = −3.9) to spatial clusters in the left inferior frontal gyrus (Table 2). The spatial component included a 135 mm^3^ cluster with negative loading in the left frontal pars orbitalis, a 324 mm^3^ cluster with positive loading in the left frontal par triangularis, and a 999 mm^3^ cluster in the left frontal opercularis with positive loading (Figure 5). This indicates that greater connectivity of the left frontal opercularis and triangularis, and less connectivity of the left frontal orbitalis, are negatively covaried with faster response time in the bound spatial and verbal working memory task.

## Discussion

Since the discovery of resting state connectivity there has been an explosion of interest in relating differences in functional networks measured at rest with behavioral or cognitive measures in both healthy and patient populations. However, to date, the majority of studies doing so have employed univariate techniques. In univariate analyses, the behavioral measure of interest is tested against (e.g., correlated with) a resting state functional connectivity measure separately at the voxel or region of interest level (ROI), with the major assumption being that each voxel, or ROI, varies independently. However, this is certainly not the case, and more powerful multivariate methods need to be established in order to find relationships between behavior and resting state functional connectivity measures.

Here, we use para-ICA to identify relationships between fluctuations in RSNs and behavior. This method has several advantages over traditional univariate methods. First and foremost, para-ICA is a multivariate method, which considers all data point and voxels within each modality simultaneously, not independently as in univariate analyses. This allows for identification of complex relationships that exist between modalities that would be missed using univariate analyses. Second, this method is completely data-driven, and no hypothesis regarding the relationship between modalities is required.

Using simulation data we first demonstrate that para-ICA results in realistic correlations between data sets with large differences in dimensionality. The validity of para-ICA on datasets with more balanced dimensions has been previously established (Liu et al., 2008). Based on our simulated data, we conclude that para-ICA performs well in the null scenario, where there is no relationship between modalities, as well as in cases with varying levels of correlations between modalities even when applied to datasets with unbalanced dimensions. This is because, under the Infomax-ICA framework, the performance of para-ICA depends on the dimensionality of the samples (e.g., subjects), and not on the dimensionality of the data modalities (Liu et al., 2008). This finding is reassuring given the data-driven nature of the method, and demonstrates that para-ICA will not induce a relationship between modalities if no real relationship exists.

In line with our primary goal, we demonstrate that common RSNs can be spatially decomposed into sub-components that covary with specific behavioral profiles. Of the nine networks we performed para-ICA on, four did not result in significant correlations between the spatial components and behavior components. As hypothesized, there were no sub-components of the auditory network that significantly covaried with any of the behavioral indices collected. This was expected, as the behavioral indices were mostly cognitive in nature, and there were no tasks with auditory information. In addition, no significantly correlated pairs of components were observed for the two lateralized attention networks and the fronto-polar network. It is interesting that the para-ICA did not identify any components between lateralized attention networks and some of the lateralized behavioral tasks. This could be related to various statistical thresholds applied in the current research. Another possible explanation is that the ICA on the behavioral measures was driven by factors other than task lateralization. However, as hypothesized, other RSNS including the default mode networks, the bilateral frontal network, and an attention network did have sub-components that covaried with components consisting primarily of specific cognitive measures. As this method is exploratory in nature, we provide limited interpretation of the results when possible.

### Temporo-parietal attention network

The para-ICA on the temporo-parietal attention network identified a spatial sub-component located in the precuneus that differentially covaried with response time in congruent and incongruent Stroop task trials. Less connectivity of this area in the precuneus to temporo-parietal attention RSN negatively covaried with better performance (faster response time) in incongruent trials, but worse performance (slower response time) in congruent trials.

The precuneus has previously been implicated in visuo-spatial processes, including attention (Beauchamp et al., 2001; Simon et al., 2002; Cavanna and Trimble, 2006). In addition, the precuneus has been shown to be preferentially activated in a color-word Stroop task with a hypothesized role of selecting task relevant information together with the dorsolateral PFC (Banich et al., 2000). Here, we show that the level of involvement of a cluster of the precuneus to the temporo-parietal attention networks has different effects on incongruent and congruent trials in the Stroop task, a task often used to measure selective attention.

### Posterior–superior default network

For the posterior–superior default network, greater connectivity from a sub-component in the cuneus was found to negatively covary with two separate working memory tasks that require the retention of spatial information. Subjects with more cuneus involvement in this RSN had slower response times in a spatial working memory task, and a combined spatial and verbal working memory task. This area of the primary visual cortex is essential for visuo-spatial processing, including working memory (for review, see Kravitz et al., 2011). In addition, previous studies have shown that the ability to suppress areas of the default mode network, including the cuneus, during working memory tasks is correlated with performance on those tasks (Hampson et al., 2006; Anticevic et al., 2010). Here we found a sub-component of the default mode network at rest that negatively covaries specifically with two tasks involving spatial working memory.

### Posterior–inferior default network

There were two sub-components of the posterior–inferior default network that covaried with specific behavioral components. The first sub-component consisted of a small cluster in the right middle occipital gyrus (BA 19) that differentially covaried with congruent Stroop task trials and a combined verbal and spatial working memory task. Greater connectivity of this area to the posterior–inferior default network positively covaried with faster response time in the working memory task and slower response time in the Stroop congruent task trials. Therefore, the connectivity of this region to this RSN has different effects on selective attention and working memory tasks.

The second sub-component of the posterior–inferior default network included a region in the vermis of the cerebellum that negatively covaried with response time to all Stroop trial types, as well as response time to incongruent Stroop trial types. Less connectivity to this RSN by the cerebellum, which has previous been implicated in Stoop task performance (Egner and Hirsch, 2005), was inversely related to slower response times in these Stroop trial types.

The identification of a sub-component of the default mode network that covaried differently to an attention task and a working memory task, and the fact that a second sub-component specifically covaried with just a selective attention task, is not without precedent. Although regions that deactivate during cognitive tasks are generally similar across task type, subtle task-dependent deactivations have been reported (Tomasi et al., 2006). Interestingly, a recent study by Mayer and colleagues found that task-induced deactivations of the default mode network differed between an attention task and a working memory task (2010). Our results are consistent with these, and suggest that the roles of specific sub-regions of the posterior–inferior default network are dependent on task-type.

### Medial frontal default network

The para-ICA on the medial frontal default network identified a spatial component consisting of the brainstem and mid-cingulate cortex that differentially covaried with two working memory tasks. The greater connectivity of the mid-cingulate and less connectivity of the brainstem to this default network was inversely related to faster response time in the verbal working memory tasks, but slower response time in a combined verbal and spatial working memory task. For the medial frontal default network, we again see regions of the default mode network that differentially respond to different tasks. However, in contrast to our findings for the posterior–inferior default network, here we identify regions of the medial frontal default network that responds differently to two working memory tasks, one involving just spatial information and one involving both verbal and spatial information. Therefore, it appears that the relationship between variations of the default mode network and cognition are not only task-dependent, but also depend on the specific division of the default network being investigated.

### Bilateral frontal network

The para-ICA performed on the bilateral frontal network resulted in a spatial sub-component that split the left inferior frontal gyrus into three separate clusters that were negatively covaried with response time in a combined spatial and verbal working memory task (bound). These three clusters included the pars opercularis (BA 44), pars triangularis (BA 45), and pars orbitalis (BA 47). Greater connectivity of pars opercularis and pars triangularis, which together are generally thought to make up Broca's Area (Keller et al., 2009), and less connectivity of pars orbitalis, was inversely related to faster response time in the combined spatial and verbal working memory task. This is especially interesting in light of the hypothesis put forward by Badre and colleagues that a dual system of cognitive control exists in the left inferior frontal gyrus (2005). According to this model, for any cognitive task requiring retrieval and selection of mnemonic information, such as a working memory task, the anterior portion of the left ventrolateral prefrontal cortex (pars orbitalis; BA 47) is responsible for the controlled retrieval of information while the mid-ventrolateral prefrontal cortex is responsible for post-retrieval selection of task-relevant representations (Badre and Wagner, 2007). Here, we show a similar dissociation between the left anterior ventrolateral prefrontal cortex (pars orbitalis) and the left mid-ventrolateral prefrontal cortex (pars opercularis and pars triangularis), as the involvement of these areas to the bilateral frontal RSN covaried differently with performance in a dual spatial and verbal working memory task.

### Limitations

This study has several limitations. One common issue with ICA analyses, including para-ICA analyses, is the number of components that are estimated. However, we believe that the 40 components estimated in the group ICA is appropriate due to the high degree of spatial correlation between our components and the common RSN templates. For the para-ICA, we based our decision by identifying the number of components that explained approximately 90% of the variance for both the behavioral data and each resting state component. Finally, this study is limited by a relatively small sample size, a larger sample size would provide more detection power, and by the fact that nine separate para-ICAs were performed. Several steps were taken in attempts to control for this issue as several different levels of the analyses (Figure 1). In addition, this study is intended as an exploratory study to show the feasibility of indentifying sub-components of RSNs that covary with specific behaviors. Accordingly, we only provide cautious interpretation of results when possible, as these findings need to be verified with other means, such as more hypothesis driven approaches, before strong interpretations can be made.

## Conclusions

In this study, we first demonstrate the utility of a para-ICA in identifying real inter-modal relationships in data with unbalanced dimensions using simulated data. We then use this simultaneous multivariate method to demonstrate that sub-components of common RSNs covary with specific behavioral measures. Importantly, the regions of these sub-components were found to covary with tasks that have previously been associated with those regions. For the temporo-parietal attention RSN, para-ICA identified a sub-component that covaried differently for two different trial types in a common sustained attention task. Likewise, for the bilateral frontal RSN, the para-ICA identified a sub-component that split the left inferior frontal gyrus into three clusters according to its cytoarchitecture that differentially covaried with performance on a working memory task. In addition, the separation of these regions of the left inferior frontal gyrus is consistent with a prominent model of dual cognitive control in the ventrolateral prefrontal cortex (Badre and Wagner, 2007). Finally, the para-ICA identified several sub-components of the default mode networks investigated that covaried differently with specific cognitive tasks, consistent with previous studies that have demonstrated task-dependent differences in deactivations of default mode regions (Mayer et al., 2010). In summary, para-ICA identified specific sub-components of common RSNs that covaried with different behavioral profiles, shedding light on the complex relationship between behavior and spontaneous brain activity.

### Conflict of interest statement

The authors declare that the research was conducted in the absence of any commercial or financial relationships that could be construed as a potential conflict of interest.
